# Neuro-vascular coupling and heart rate variability in patients with type II diabetes at different stages of diabetic retinopathy

**DOI:** 10.3389/fmed.2022.1025853

**Published:** 2022-11-10

**Authors:** Nikolaus Hommer, Martin Kallab, Andreas Schlatter, Patrick Janku, René M. Werkmeister, Kinga Howorka, Doreen Schmidl, Leopold Schmetterer, Gerhard Garhöfer

**Affiliations:** ^1^Department of Clinical Pharmacology, Medical University of Vienna, Vienna, Austria; ^2^Hanusch Hospital, Karl Landsteiner Institute, Vienna, Austria; ^3^Hanusch Hospital, Vienna Institute for Research in Ocular Surgery, Vienna, Austria; ^4^Center for Medical Physics and Biomedical Engineering, Medical University of Vienna, Vienna, Austria; ^5^Metabolic Competence Center, Medical University of Vienna, Vienna, Austria; ^6^Singapore National Eye Centre, Singapore Eye Research Institute, Singapore, Singapore; ^7^Ophthalmology and Visual Sciences Academic Clinical Program, Duke-NUS Medical School, Singapore, Singapore; ^8^School of Chemical and Biomedical Engineering, Nanyang Technological University, Singapore, Singapore; ^9^Institute of Clinical and Experimental Ophthalmology, Basel, Switzerland

**Keywords:** diabetes type II, diabetic retinopathy, functional hyperemia, neuro-vascular coupling, retinal blood flow, heart rate variability

## Abstract

**Aims/Hypothesis:**

There is evidence that diabetes is accompanied by a break-down of functional hyperemia, an intrinsic mechanism of neural tissues to adapt blood flow to changing metabolic demands. However, to what extent functional hyperemia is altered in different stages of diabetic retinopathy (DR) in patients with type II diabetes is largely unknown. The current study set out to investigate flicker-induced retinal blood flow changes in patients with type II diabetes at different stages of DR.

**Materials and methods:**

A total of 76 subjects were included in the present parallel-group study, of which 56 had diabetes with either no DR or different stages of non-proliferative DR (*n* = 29 no DR, 12 mild DR, 15 moderate to severe DR). In addition, 20 healthy subjects were included as controls. Retinal blood flow was assessed before and during visual stimulation using a combined measurement of retinal vessel calibers and blood velocity by the means of Doppler optical coherence tomography (OCT). To measure systemic autonomic nervous system function, heart rate variability (HRV) was assessed using a short-term orthostatic challenge test.

**Results:**

In healthy controls, retinal blood flow increased by 40.4 ± 27.2% during flicker stimulation. Flicker responses in patients with DR were significantly decreased depending on the stage of the disease (no DR 37.7 ± 26.0%, mild DR 26.2 ± 28.2%, moderate to severe DR 22.3 ± 13.9%; *p* = 0.035, ANOVA). When assessing systemic autonomous neural function using HRV, normalized low frequency (LF) spectral power showed a significantly different response to the orthostatic maneuver in diabetic patients compared to healthy controls (*p* < 0.001).

**Conclusion/Interpretation:**

Our study indicates that flicker induced hyperemia is reduced in patients with DR compared to healthy subjects. Further, this impairment is more pronounced with increasing severity of DR. Further studies are needed to elucidate mechanisms behind the reduced hyperemic response in patients with type II diabetes.

**Clinical trial registration:**

[https://clinicaltrials.gov/], identifier [NCT03 552562].

## Introduction

The incidence and prevalence of type II diabetes and its associated co-morbidities is constantly rising (1–3). Traditionally, diabetic complications are classified as macrovascular – leading to cardiovascular and peripheral vascular disease – and microvascular complications, the latter being characterized by the triade of nephropathy, neuropathy, and diabetic retinopathy (DR) (4). Although microvascular damage usually occurs early in the disease process, changes can be clinically silent for a long time before a symptomatic manifestation occurs. Thus, biomarkers of disease progress reflecting microvascular complications are still warranted to identify high-risk patients (5).

The eye has the unique advantage that the microvasculature can be assessed directly and non-invasively without the need for an injection of contrast agents or radiation exposure. As such, microvascular changes such as microaneurysms, hemorrhages, or hard exudates are well known clinical signs of DR that can easily be graded on the slit lamp or using fundus photography. Further, recently developed imaging devices such as optical coherence tomography (OCT) angiography show rarefication of retinal vessels (6–10) indicative for changes in retinal perfusion (11–15).

However, there is evidence that functional alterations may proceed structural changes in patients with diabetes. As such, it has been consistently shown in the brain as well as in the retina that functional hyperemia, a term describing the ability of the tissue to adapt blood flow to different metabolic demands is impaired in patients with diabetes. However, whereas this hyperemic response, also known as neuro-vascular coupling is difficult to investigate in the brain and requires costly investigations such as Positron emission tomography (PET) or functional magnetic resonance imaging (fMRI) (16–18), neuro-vascular coupling can be easily and none-invasively assessed at the level of the retina by visual stimulation with flickering light. As such, most (19–26) but not all (27) human studies indicate a breakdown of neuro-vascular coupling in patients with diabetes. However, previous studies on this topic were limited by the fact that only information regarding vessel diameter not volumetric blood flow was assessed. Thus, the retinal blood flow response to flicker stimulation in patients with type II diabetes is largely unknown (24).

The present study set out to extend our knowledge on neuro-vascular coupling in patients with type two diabetes and different stages of DR. To assess retinal blood flow under visual stimulation, a recently developed, custom-built Doppler OCT system was used. Based on the measurement of retinal vessel diameters and blood flow velocity our approach allows us to expand our knowledge and assess volumetric blood flow during visual stimulation (28, 29). In addition, the current study included a test for measuring autonomic nervous system function. This test assesses heart rate variability (HRV) (30–32) and allows to investigate whether alterations in the autonomic nervous system may correlate with the severity of DR.

## Materials and methods

### Subjects

The study protocol was approved by the Ethics Committee of the Medical University of Vienna, the national competent authorities and was conducted in compliance with the Declaration of Helsinki and Good Clinical Practice (GCP) guidelines of the European Union. All subjects, selected by the Department of Clinical Pharmacology, provided written informed consent before any study-related procedures were performed. All subjects passed a prestudy screening in the 4 weeks before the first study day including the following examinations: medical history, pregnancy test in women of childbearing potential, blood pressure and heart rate measurement, laboratory testing for HbA1c, blood glucose levels and hemoglobin, assessment of visual acuity, slit lamp biomicroscopy, indirect funduscopy, 7-standard field color fundus photography for grading of DR (33), and measurement of intraocular pressure (IOP) with Goldmann applanation tonometry. Subjects were not included, if any clinically significant medical condition (except diabetes type II in the patient group) was found as part of the screening examination. Exclusion criteria were ametropia of more than six diopters, smoking and history, or family history of epilepsy.

### Study design

The current study was a prospective parallel group study with observer blinded analysis.

On the screening day and on the study day all subjects arrived in the morning after 7–8 h of sleep and abstained from alcohol and stimulating beverages containing xanthine derivates like tea, coffee, or cola-like drinks 12 h before the study day. At the beginning of the study day, a pregnancy test was performed in females of childbearing potential. Then, one drop of mydriaticum (Mydriaticum “Agepha,” Agepha, Vienna, Austria) was administered into the chosen study eye and a resting period of at least 20 min was scheduled before measurements were started to allow pupil dilatation and ensure constant hemodynamic conditions. Capillary blood glucose level measurements were performed using a glucose meter and a blood draw for laboratory testing for HbA1c was done. Then, retinal vessel diameters were measured at baseline and during flicker stimulation using the dynamic vessel analyzer (DVA) and retinal venous blood flow using DOCT was assessed. Blood pressure, heart rate, and IOP were measured before and after these measurements. Assessment of HRV was done as final study day investigation. Blood flow measurements were performed under light-adapted conditions.

### Measurement of retinal vessel diameters

Measurement with the DVA allows the real time determination of retinal vessel diameters *in vivo*. This commercially available system (IMEDOS, Jena, Germany) combines a fundus camera (FF 450; Carl Zeiss Meditec, Jena, Germany), a video camera, a real time monitor and a personal computer with an analyzing software to measure retinal and venous diameters (34). The device allows the evaluation of the diameters of one temporal retinal artery and vein between 1 and 2 disk diameters from the margin of the optic disk by analyzing digital pictures from a continuous video of the respective vessels (35).

Before flicker stimulation, a baseline recording for 60 s was performed. After the baseline recording, flicker was applied for another period of 60 s. For flicker stimulation, the white light spectrum of a halogen lamp was filtered using a 550 nm low pass cut-off-filter, ensuring that the yellow and red spectral part are filtered and only wavelengths below 550 nm were used for stimulation. Flicker was applied at a frequency of 12.5 Hz.

### Measurement of retinal venous blood flow

A previously described system allows quantification of retinal venous blood flow *in vivo* (13, 28, 29, 36–38). This custom-built dual-beam Doppler Fourier Domain-OCT (DOCT), coupled to a fundus camera, which ensures simultaneous view for selection of the region of interest, used a specific rectangular scanning pattern around the optic nerve head (ONH) to gain measurements from retinal veins with a diameter of at least 40 μm. To obtain averaged blood velocity values, this scanning pattern was applied on each vessel for several pulse periods. For quantification of mean absolute blood velocity (*V*_*abs*_), phase shifts in the two probe beams and channels, respectively, were calculated. Venous blood flow *(Q*_*abs*_*)* was calculated using vessel diameters *(d)*, which were extracted from the DVA and *V*_*abs*_ as *Q*_*abs*_ = *V*_*abs*_*d*_2_ π/4 for each vessel. DVA and DOCT measurements were performed simultaneously at the same vessel location. The detailed description of the setup has been published previously (28).

### Measurement of heart rate variability

Measurement of HRV is a standardized, non-invasive method for quantification of autonomic sympathetic and parasympathetic control and therefore used for evaluation of cardiovascular autonomic neuropathy in diabetes (39–41). This assessment is based on analysis of HRV in frequency-domain, using a short-term modified orthostatic load (42). Using VariaCardio^®^ (Vario Cardio TF5; Advanced Medical Diagnostic Group, Leeds, UK), this short-term spectral analysis of HRV is obtained from recordings consisting of 256 s of artifact-free records in three positions (supine1-standing-supine2). The recorded high-resolution one-channel echocardiogram (ECG) is transferred to a receiver connected to a personal computer and displayed on-line. Then R–R intervals are identified with a sampling rate of 1,000 Hz. Artifacts are identified and labeled and a specific algorithm inserts beat-to-beat intervals throughout an artifact period to preserve the timing relationships of the adjacent, uncorrupted heart rate data. The final results are immediately displayed on the monitor as three-dimensional running spectra. Parameters of frequency-domain HRV are measured within the low-frequency (LF) band (0.05–0.15 Hz) and high-frequency (HF) band (0.15–0.50 Hz). A reduced LF and HF spectral power at rest, a reduced increase of normalized LF spectral power during orthostasis and a reduced normalization after an orthostatic challenge are known to be a sensitive sign of a limited autonomic cardiovascular control (42, 43).

### Statistical analysis

Statistical analysis was performed using IBM SPSS Statistics (Version 26, IBM, Armonk, NY, USA). All values are presented as means ± SD. Normal distribution for all outcome variables was confirmed using the Shapiro–Wilk test. Extreme outliers [according to the 3 (IQR) rule], were removed, these were two data points for the flicker response in arterial diameter in the “no DR” group. Descriptive statistics are reported for all values obtained. Flicker response was calculated as percent change from baseline in vessel diameter and retinal blood flow. A Welch ANOVA was carried out for all parameters to assess overall differences between groups. Planned contrasts between groups were used to assess differences between two groups (in particular between healthy subjects, patients with no DR, patients with mild DR, and patients with moderate to severe DR). To assess differences in HRV during the orthostatic maneuver, a repeated measures ANOVA model was calculated. A *p*-value < 0.05 was considered as the level of significance.

## Results

A total of 76 subjects were included in the present study, of which 56 had diabetes with no DR or different stages of non-proliferative DR. Among these, twenty-nine (29) had no DR, 12 had mild DR, and 15 had moderate to severe DR. In addition, 20 ages matched healthy subjects were included as controls. The demographics and baseline characteristics of the four study groups are shown in Table 1.

**TABLE 1:** Baseline characteristics of the four study groups.

	Healthy subjects (*n* = 20)	No DR (*n* = 29)	Mild DR (*n* = 12)	Moderate to severe DR (*n* = 15)
Age (years)	59 ± 9	61 ± 10	64 ± 9	63 ± 7
Body mass index (kg/m^2^)	26 ± 6	28 ± 5	30 ± 3	30 ± 5
Diabetes duration (years)	N/A	14 ± 10	15 ± 10	11 ± 7
HbA1c (%)	N/A	6.6 ± 0.7	6.9 ± 0.8	7.3 ± 1.0
Plasma glucose level (mg/dL)	N/A	184 ± 58	186 ± 63	221 ± 52
Systolic blood pressure (mmHg)	128 ± 13	135 ± 13	130 ± 15	138 ± 11
Diastolic blood pressure (mmHg)	77 ± 10	80 ± 11	76 ± 14	77 ± 10
Heart rate (bpm)	69 ± 8	71 ± 12	72 ± 10	71 ± 10
Mean arterial pressure (mmHg)	100 ± 12	104 ± 11	100 ± 13	106 ± 9
Intraocular pressure (mmHg)	14 ± 2	15 ± 3	15 ± 2	15 ± 2
Ocular perfusion pressure (mmHg)	52 ± 7	55 ± 8	52 ± 8	55 ± 7
Retinal venous blood flow obtained in one single vessel (μl/min)	15.7 ± 6.3	15.0 ± 9.4	17.8 ± 4.0	13.5 ± 6.2

Values are presented as mean ± SD.

**FIGURE 1:**
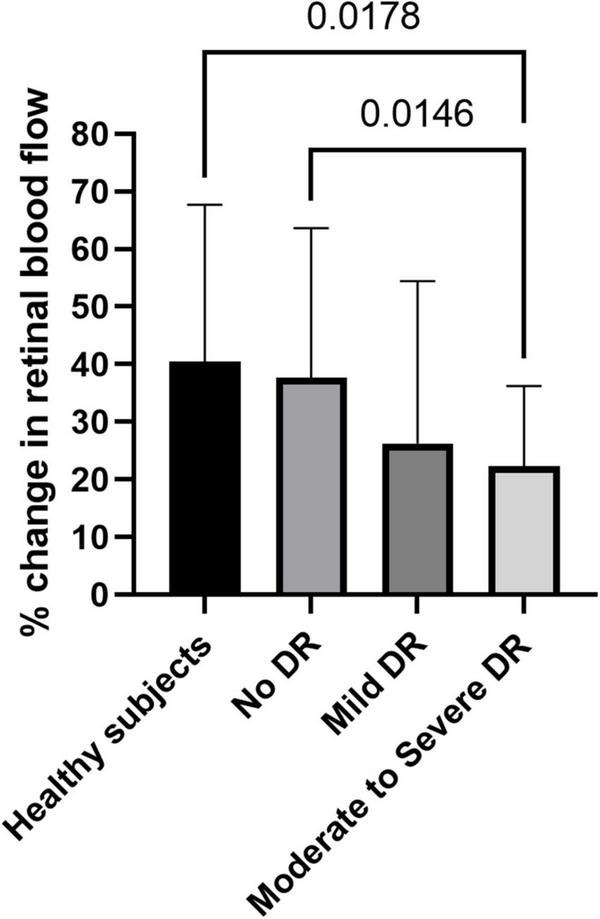
Flicker response of retinal blood flow in healthy subjects, patients with no diabetic retinopathy (no DR), mild diabetic retinopathy (mild DR), and moderate to severe diabetic retinopathy (moderate to severe DR). Data are presented as mean ± SD. Planned comparisons were used to calculate *p*-values between groups.

A significantly different response to flicker stimulation was observed between the four groups in regards to retinal venous blood flow (Figure 1). While in healthy subjects, retinal venous blood flow increased by 40.4 ± 27.2% and in patients with no DR by 37.7 ± 26.0%, it only increased by 26.2 ± 28.2% in patients with mild DR, and by 22.3 ± 13.9% in patients with moderate to severe DR (*p* = 0.035).

**FIGURE 2:**
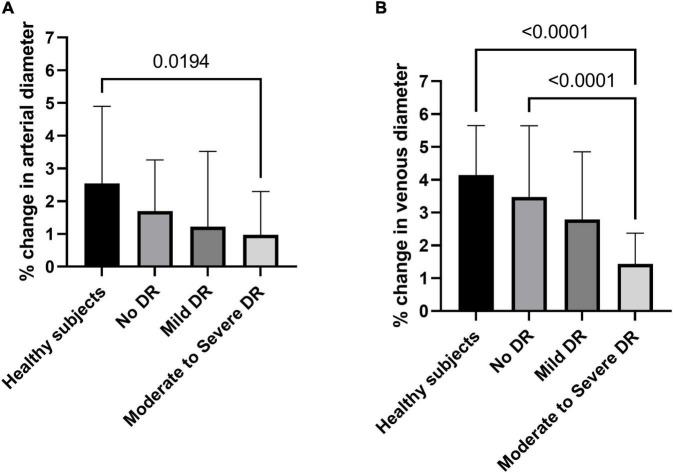
Flicker response of retinal arterial **(A)** and venous **(B)** diameter in healthy subjects, patients with no diabetic retinopathy (no DR), mild diabetic retinopathy (mild DR), and moderate to severe diabetic retinopathy (moderate to severe DR). Data are presented as mean ± SD. Planned comparisons were used to calculate *p*-values between groups.

The hyperemic response in retinal arterial diameter was 2.5 ± 2.4% in healthy subjects, 1.7 ± 1.6% in patients with no DR, 1.2 ± 2.3% in patients with mild DR, and 1.0 ± 1.3% in patients with moderate to severe DR, respectively. Although there was a tendency toward a decreased response in arterial diameters with increasing stage of DR, this effect failed to reach significance (*p* = 0.109, Figure 2A). Planned comparisons revealed that there was a significant difference in arterial diameter response between healthy subjects and patients with moderate to severe DR.

As for retinal veins, the response of venous diameters to flicker light was significantly different between the four groups (*p* < 0.001, Welch ANOVA, Figure 2B). Flicker induced vasodilation was 4.1 ± 1.5% in healthy subjects, 3.5 ± 2.2% in patients with no DR, 2.8 ± 2.1% in patients with mild DR, and lowest in patients with moderate to severe DR (1.4 ± 0.9%). When comparing individual groups, the response was significantly different between patients with moderate to severe DR and healthy subjects or patients with no DR.

**FIGURE 3:**
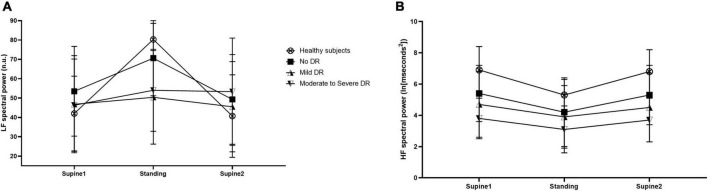
Normalized low-frequency (LF) spectral power of heart rate variability (HRV) in normalized units (nu) **(A)** and high-frequency (HF) spectral power **(B)** in healthy controls (open circles), patients with no diabetic retinopathy (no DR) (black squares), patients with mild diabetic retinopathy (mild DR) (upward triangles), and patients with moderate to severe diabetic retinopathy (moderate to severe DR) (downward triangles) before, during and after the orthostatic challenge. Data are presented as mean ± SD.

When looking at HRV, normalized LF spectral power showed a significantly different response to the orthostatic maneuver in diabetic patients compared to healthy controls (*p* < 0.001). As shown in Figure 3A, the response was more blunted with increasing severity of DR. Data showed a significant difference between healthy subjects and patients with no DR (*p* = 0.004), healthy subjects and patients with mild DR (*p* < 0.001) and healthy subjects and moderate to severe DR (*p* < 0.001).

The time course of HF spectral power during the orthostatic maneuver was not altered in diabetic patients compared to healthy controls (*p* = 0.270, Figure 3B). However, HF spectral power was generally lower at all assessments in patients with diabetes compared to healthy subjects. No correlation between the flicker response in retinal blood flow, retinal arterial diameter, retinal venous diameter, and the HRV parameters normalized LF and HF spectral power was found (*p* > 0.30 each).

## Discussion

The results of the present study indicate that the blood flow response to visual stimulation with flicker light is reduced with increasing severity of DR in patients with type II diabetes. This effect was paralleled by an altered HRV in patients with diabetes, again associated with disease severity. In summary, our data support the concept of a strong neuro-vascular component in the pathogenesis of DR in patients with type II diabetes.

Several lines of evidence indicate that in the brain, as well as in the retina, a breakdown of neuro-vascular coupling may be a pathogenic factor in the development of diabetic complications. Studies in the brain using blood oxygenation level–dependent functional magnetic resonance imaging (BOLD fMRI) to assess functional hyperemia have shown reduced functional hyperemia in patients with diabetes (44, 45). As for the retina, there is compelling evidence that flicker induced vasodilatation is reduced in patients with type I and type II diabetes (20, 23, 46–48). However, the latter studies do not give a full picture of functional hyperemia in human diabetic subjects: First, previous studies report only data regarding flicker induced retinal vasodilatation in large retinal vessels, but not blood flow *per se*. Given that the microcirculation is the major determinant of vascular resistance and therefore blood flow, caliber changes in large vessel do not necessarily fully predict changes in blood flow. Secondly, studies assessing neuro-vascular coupling in different clinical stages of DR are sparse. Mandecka et al. (46) have reported reduced retinal vasodilatation, an effect that was more pronounced in more severe patients. As the latter study has included patients with both type I and type II diabetes, it remains unclear whether type I and type II diabetics share the same characteristics in neuro-vascular coupling.

Our data expands our knowledge in patients with type II diabetes and indicates that flicker-induced hyperemia is reduced in patients with moderate to severe DR compared to healthy subjects. Indeed, the results show that in patients with moderate to severe non-proliferative DR, blood flow response is approximately only 50% of the response observed in the healthy control group. Further, the reduction in flicker induced hyperemia was also significantly more pronounced in patients with moderate to severe DR compared to patients with no or mild DR indicating that neuro-vascular coupling is reduced depending on the severity of DR. Thus, our data supports the hypothesis of a break-down in neuro-vascular coupling early in the disease processes, which may aggravate with the progression of the disease. They also extend our previous findings in patients with type I diabetes, showing that the blood flow response to flicker stimulation is significantly reduced (22). However, since in this previous study only patients with no DR were included, no conclusion can be made on a potential difference between different grades of severity.

The reason for the diminished hyperemic response in patients with type II diabetes is still not fully elucidated. It has been suggested that the uncoupling between neural function and blood flow in diabetes may be related to endothelial dysfunction (23, 46). Following this hypothesis, the impaired hyperemic response is related to an impaired dilatory response of the microvasculature. Indeed, an impaired endothelial-dependent vasodilatation has been demonstrated in other vascular beds such as the brachial artery (49). Further, it has been reported that conditions associated with endothelial dysfunction such as smoking or systemic hypertension are also characterized by diminished flicker induced vasodilatation (47, 50). On the other hand, it has been shown that the vasodilatory response of retinal vessels to exogenous nitric oxide is preserved in patients with diabetes, indicating that the impaired flicker response is not caused by a generally reduced retinal vascular reactivity of retinal vessels (51). Thus, the question whether reduced flicker response can mainly be attributed to endothelial dysfunction is not entirely clear.

Alternatively, it has been hypothesized that impaired neural activity may be responsible for the observed uncoupling of visual stimulation and blood flow (52). Along this line of thought it has consistently been demonstrated that both the retinal nerve fiber layer as well as the ganglion cell layer are considerably thinner in patients with diabetes and no DR as compared to healthy controls (53, 54). This observed retinal thinning is associated with HbA1c, duration of diabetes and the severity of DR, indicating that neural degeneration plays an important role in the pathogenesis of the disease (55, 56). Whether these neurodegenerative changes observed in patients with diabetes occur prior to vascular manifestations or develop independently from them is yet to be investigated (52). Finally, longitudinal trials are warranted to clarify whether neural dysfunction plays a causative role in the observed breakdown of neuro-vascular coupling.

Impairment of neural function is also reflected in the data of the current study. In particular, differences between the four groups were also found for HRV, a marker for the autonomic nervous system (31, 32). While HF spectral power reflects vagal activity, the LF component is interpreted as a marker for sympathetic modulation (31). LF spectral power showed a statistically significant different response to the orthostatic maneuver in all patients with diabetes compared to healthy subjects and the difference became more pronounced with increasing disease severity. This all points toward dysfunction of the autonomic system in patients with type II diabetes. Along this line of thought, several studies report disturbed function of the autonomic nervous system as a risk factor for cardiovascular disease (57, 58), which also has a higher prevalence in diabetic patients (59, 60). Interestingly, there also seems to be a link between the autonomic nervous system and the vascular endothelium in that sense that both act in opposition in order to regulate vascular tone (61). However, as the retinal blood supply is not under direct autonomic nerve control, a potential effect of the observed dysfunction of the autonomic system in patients with type II diabetes on ocular blood flow needs to be further investigated.

Some strengths and limitations of the present study need to be addressed. The strength of the current study is that by using the Doppler OCT technique, retinal vessel diameter and blood flow could be measured concomitantly, which allows for the exact determination of volumetric blood flow. We have recently shown that this system provides excellent repeatability and reproducibility in detecting even subtle changes in retinal blood flow (29). However, as a disadvantage of the used approach, the measurements during visual stimulation are challenging for the subjects and require excellent target fixation. Thus, blood flow measurement data have only been obtained from one vessel and no information on total retinal blood flow is available from the current study. In this context, it needs to be noted that there is still no general agreement in the literature on blood flow changes in patients with diabetes. Whereas some studies found decreased retinal perfusion in patients with diabetes (11, 12, 62, 63), others have reported an increase, especially in early stages (13–15, 64). Thus, further studies are needed to clarify this issue. Further, it also needs to be mentioned that some of the patients were under systemic antihypertensive medication and we cannot fully exclude that this may have influenced ocular perfusion parameters. However, as the outcome of the study was flicker induced hyperemia not absolute blood flow and systemic hemodynamic procedures were comparable between the study days, we deem that this does not interfere with our conclusions.

An additional limitation of the current study is the cross-sectional design, which allows only for conclusions regarding one certain time point, but does not finally clarify whether impaired neuro-vascular coupling is causative for the development of retinal vascular complications of diabetes type II. Longitudinal studies are required to further investigate this issue.

## Conclusion

In conclusion, the results of the present study show that retinal neurovascular coupling and HRV are both impaired in patients with type II diabetes. These impairments seem to be more pronounced in later stages of DR. Further studies are required to assess whether the break-down of flicker induced hyperemia may be used as a predictive marker for the individual risk assessment of DR.

## Data availability statement

The raw data supporting the conclusions of this article will be made available by the authors, without undue reservation.

## Ethics statement

The studies involving human participants were reviewed and approved by the Ethics Committee of the Medical University of Vienna. The patients/participants provided their written informed consent to participate in this study.

## Author contributions

NH, KH, DS, LS, and GG: conceptualization. NH, RW, LS, and GG: methodology. RW: software. NH, DS, and GG: validation, formal analysis, and visualization. NH, MK, AS, PJ, and GG: investigation. KH and GG: resources. DS and GG: data curation. NH and GG: writing – original draft preparation and project administration. MK, AS, PJ, RW, KH, DS, and LS: writing – review and editing. RW and GG: supervision. GG: funding acquisition. All authors contributed to the article and approved the submitted version.
